# Measurement of Salivary Cortisol in Two New World Primate Species

**DOI:** 10.3390/biology12091181

**Published:** 2023-08-29

**Authors:** Martina Stocker, Eoin P. O’Sullivan, Rupert Palme, Eva Millesi, Ruth Sonnweber

**Affiliations:** 1 Animal Science Department, Biomedical Primate Research Centre, 2288 GJ Rijswijk, The Netherlands; 2 Department of Behavioral and Cognitive Biology, University of Vienna, 1030 Vienna, Austria; 3 School of Psychology and Neuroscience, University of St Andrews, St Andrews KY16 9JP, UK; 4 Unit of Physiology, Pathophysiology and Experimental Endocrinology, Department of Biomedical Sciences, University of Veterinary Medicine, 1210 Vienna, Austria

**Keywords:** HPLC, enzyme immunoassay, saliva swab, glucocorticoid, steroid hormone, capuchin monkey, squirrel monkey, *Sapajus* spp., *Saimiri sciureus*

## Abstract

**Simple Summary:**

Glucocorticoids (GCs) play a vital role in the body’s response to stress. Therefore, scientists use GC level measurements as a valuable tool to understand the well-being of animals in behavioral, conservation, and welfare research. GC levels can be measured from different sample types, one of them being saliva. Saliva samples are especially useful because they can be collected rather easily and in a non-invasive manner. The aim of this study is to validate a method to measure salivary GCs, i.e., an enzyme immunoassay, in two monkey species: capuchin monkeys and squirrel monkeys. Individuals of both species were trained to provide saliva samples. Using high-pressure liquid chromatography, we confirmed that two main GCs, cortisol, and cortisone, are present in the saliva of these species and that they can be detected by the enzyme immunoassay. We further verified the biological validity of the assay in the two species by showing that it can track the decrease in cortisol levels throughout the day, which is typical in these monkeys. Our findings support the usefulness of the enzyme immunoassay to measure salivary cortisol levels in these non-human primate species and highlight its potential as a valid tool in research and welfare assessment.

**Abstract:**

Glucocorticoids (GCs) are mammalian steroid hormones involved in a variety of physiological processes, including metabolism, the immune response, and cardiovascular functions. Due to their link to the physiological stress response, GC measurement is a valuable tool for conservation and welfare assessment in animal populations. GC levels can be measured from different matrices, such as urine and feces. Moreover, especially in captive settings, measuring GCs from saliva samples proved particularly useful as those samples can be collected non-invasively and easily from trained animals. Salivary GC levels can be measured using a variety of analytical methods, such as enzyme immunoassays. However, it is crucial to validate the analytical method for each specific application and species when using a new matrix. Using high-pressure liquid chromatography and a cortisol enzyme immunoassay, we show that the main glucocorticoids secreted in the saliva of squirrel monkeys and brown capuchin monkeys are cortisol and cortisone. Our biological validation found the expected salivary cortisol level to decline throughout the day. Our findings support the reliability of salivary cortisol measurements and their potential to be used as a valid tool in research and welfare assessment for these non-human primates.

## 1. Introduction

Glucocorticoids (GCs) are steroid hormones involved in a wide range of vital physiological processes in mammals [1]. In behavioral, welfare, and conservation research, monitoring changes in GC levels have received attention due to their involvement in the physiological stress response (e.g., [2,3]) and their association with emotional states (e.g., [4]). When the hypothalamic–pituitary–adrenal (HPA) axis is stimulated by a stressor that threatens homeostasis, cortisol (the main GC in many mammals) is released into the bloodstream to restore it [3]. Therefore, an elevation in cortisol levels indicates a threat to homeostasis and the organism’s effort to restore it [5,6]. This adaptive process of maintaining homeostasis and the accompanying changes in cortisol levels can be activated by a multitude of diverse factors, inclsuding food intake, activity patterns (such as increased movement), mating; and external stressors like extreme ambient temperature, noise exposure, and social conflicts [7]. Additionally, cortisol levels are influenced by life-history factors, such as reproductive periods and events; as well as environmental factors like season [8,9,10,11]. Furthermore, pleasurable experiences can also cause an increase in cortisol levels [12].

Cortisol levels in the blood follow a typical fluctuation pattern over a 24 h cycle, known as the diurnal rhythm of cortisol. In species that are active during the day, cortisol levels peak in the morning, shortly after waking up, and then decrease rapidly in the hours that follow. During the first few hours of sleep, blood cortisol levels reach their lowest point and remain low throughout the night [13,14,15,16]. This diurnal pattern is observed in various diurnal species, including horses [17]; cetaceans (bottlenose dolphins and orcas [18]); elephants [19]; and apes such as bonobos [20]. It can be detected not only in blood but also in other body fluids, such as saliva (e.g., [17,20,21]) and urine [19]. This rhythmic pattern has been associated with the functioning of various processes, including sleep-wake patterns [22], metabolism, learning, and memory [23]; and immune functions [24]. Due to the involvement of GCs, their measurement has become a crucial conservation and welfare assessment tool for animals [25].

Changes in the levels of cortisol metabolites can be monitored in different organic matrixes, such as urine and feces [26]. Non-invasive methods of GC measurement have been developed and validated as invasive blood sampling can be a stressor to the animal and sometimes impractical in the field [26]. The analysis of cortisol level changes from saliva samples is particularly convenient in captive settings as individuals can easily be trained to provide saliva samples [27,28]. Furthermore, this method allows frequent sampling of the same individual in fast succession. Moreover, salivary cortisol levels are highly correlated with serum cortisol concentration at a given time point [13,29,30].

For the assessment of steroid hormone concentration in organic substrates (saliva, milk, urine, feces, hair, and feathers), many excellent methods, such as enzyme immunoassays (EIAs) and mass spectrometry [31] are available (e.g., reviewed in [32,33,34]). Differences in biochemical and structural characteristics of samples between substrates and species can have major implications for the suitability of an existing immunoassay to measure steroid hormone levels in a new substrate or species [35,36]. First, depending on sample type and species, the target molecule may differ; for instance, the primary glucocorticoid found in blood or saliva of many mammalian species is cortisol (e.g., [37]). In the feces of these species, native cortisol is usually not found (in large quantities) and only metabolized forms of glucocorticoids can be measured [38]. Thus, an immunoassay with appropriate binding characteristics that may differ depending on the sample type has to be selected. Secondly, other, non-target molecules in a sample may bind to the antibody (cross-reaction), which would lead to false results for the target hormone. Hence, before using an immunoassay for a new substrate and/or species, molecules that may cross-react with the binding agents have to be identified [39]. Thirdly, species may differ in how they metabolize and excrete hormones [38]. Examining which hormones or hormone metabolites should be targeted by an assay for a given sample substrate and species is of utmost importance to render biologically meaningful results. To summarize, it is crucial to validate EIAs when applied to new species and sample types to ensure accurate and reliable GC measurements [26,34,35,40].

Capuchin monkeys (*Cebus* spp., *Sapajus* spp.) and squirrel monkeys (*Saimiri* spp.), both belonging to the family Cebidae, are intensively studied in biomedical research, neuroscience, cognitive biology, or metabolic disorders [41,42,43,44]. Furthermore, capuchin monkeys and squirrel monkeys are often exhibited in zoos. Therefore, assessment of GC levels as a metabolic or stress-related marker may be valuable. In capuchin monkeys, cortisol and/or its metabolites has already been measured in hair [45] and feces [46,47]. However, to our knowledge, there are no peer-reviewed studies published which measure salivary cortisol in capuchin monkeys. In squirrel monkeys, at least six studies have investigated salivary cortisol levels so far [48,49,50,51,52,53]. Most of those studies are based on a rather small sample size and only a single study reported having measured salivary cortisol levels in a female individual (one male and one female, [48]); the remaining published work has either only reported measurements in males or the sex has been unreported [49,50,51,52,53].

To promote studies using cortisol measurements gained from non-invasively collected saliva samples in these two non-human primate species that are often kept under human care, the aim of the current study was to validate an in-house cortisol EIA (antibodies against cortisol-3-CMO:BSA [54]). Therefore, we collected saliva samples from male brown capuchin monkeys (*Sapajus* spp.) and a group of female squirrel monkeys (*Saimiri sciureus*). We performed a high-performance liquid chromatography (HPLC) to confirm that the target molecule cortisol was indeed present in the saliva of both species and could therefore be detected by the chosen cortisol EIA and to detect molecules that may cross-react with the assay antibodies. To assess the reliability of cortisol measurements, we examined parallelism of serially diluted pooled saliva samples and the standard curves of the assay for each species. Finally, we quantified cortisol concentrations from a of total 455 saliva samples (114 capuchin monkey samples and 341 squirrel monkey samples) with the chosen cortisol EIA and tested the biological validity by investigating whether salivary cortisol concentrations decreased throughout the day, as would be predicted by the circadian cortisol secretion pattern shown in blood for both species (capuchin monkeys [55], squirrel monkeys [56]).

## 2. Materials and Methods

### 2.1. Animals and Housing

Saliva samples were collected from 17 female squirrel monkeys, *Saimiri sciureus* (mean age = 6.69 years ± 2.62 SD) and 8 male brown capuchin monkeys, *Sapajus* ssp. (mean age = 5.19 years ± 2.02 SD) housed at the Living Links to Human Evolution Research Centre at Edinburgh Zoo, UK. The two species were co-housed in the same enclosures and thus lived in two mixed-sex and mixed-species groups, the “East group” and the “West group”. At the time of testing the East group consisted of 13 capuchin monkeys (6 females and 7 males) and 19 squirrel monkeys (15 females and 2 males). The West group consisted of 16 capuchin monkeys (7 females, 7 males, and 2 unsexed infants) and 16 squirrel monkeys (14 females and 2 males). Each group had an indoor (31.50 m^2^, 12 h light-dark cycle 07:30–19:30) and outdoor area (approx. 900 m^2^) shared by all individuals from each group. Additionally, the squirrel monkeys from both groups had an indoor area (24.75 m^2^) which was not accessible for the capuchin monkeys. Both species received main feeds in all indoor areas twice a day with a mix of vegetables and fruit [57]. Three to four additional scatter feeds were provided at variable times throughout the day and water was available ad libitum.

### 2.2. Saliva Collection

Using positive reinforcement training, monkeys were trained to chew on an oral swab (Salimetrics, SalivaBio Children’s Swab) that was presented to them by the experimenter. Once an individual was able to provide saliva samples, data collection for the project started. Samples were collected within one minute; put in a labelled plastic vial; placed immediately on ice packs; and stored in a freezer at −20 °C within one hour after sample collection. To avoid hormonal contamination, the monkeys were trained not to touch the swabs with their hands and the experimenters wore gloves.

**Figure 1:**
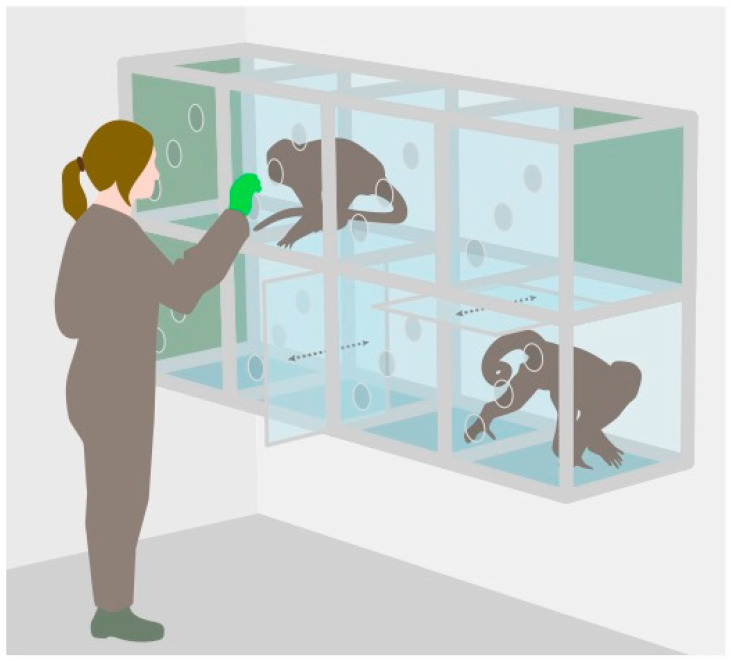
Research cubicles at Living Links Research Centre, Edinburgh Zoo. The green squares on the left indicate the exits towards the outdoor enclosure and on the right towards the indoor enclosure. All exits can be closed with sliding doors. Between each cubicle, partitioning slides can be inserted to separate individual monkeys.

Saliva samples of the squirrel monkeys were collected between 28 January 2013 and 16 December 2013. The capuchin monkey sample collection took place between 16 September 2013 and 9 December 2013. All samples were collected between 11:00 and 13:00 and between 14:00 and 16:00, as the study aimed to investigate the daily pattern of cortisol. Data collection times at Edinburgh Zoo were restricted, which did not allow for sample collection to cover the complete circadian cortisol excretion pattern. Saliva collection was conducted in separate research cubicles (49.5 cm × 52.1 cm × 51.4 cm; separate ones for each species) arranged in a 2 × 4 matrix that created a corridor connecting the indoor and outdoor enclosure (see Figure 1). Partitioning slides were inserted between cubicles to separate individual monkeys from their groupmates—a procedure these individuals were familiar with. Once an individual was isolated in a research cubicle and all other monkeys had left; the entrances to the cubicles were closed; and the saliva sample collection started; typically taking less than one minute. Participation of the monkeys was strictly voluntary. If an individual showed clear signs of discomfort, such as persistently touching the door leading to its enclosure, prior to or during saliva collection, the individual was promptly released and the saliva collection session was terminated.

### 2.3. Salivary Cortisol Analyses

Samples of both species were transported from Edinburgh to Vienna on dry ice overnight. In February 2014, the samples were analyzed in the Endocrinology Laboratory of the Department of Behavioural Biology, University of Vienna, Austria. Prior to analysis, samples were stored frozen at −20 °C. In the laboratory, the saliva swabs were thawed, followed by centrifugation (3600 rpm, 10 min). The resulting saliva was then diluted with assay buffer using predetermined (through serial dilution of pooled samples) ratios (squirrel monkeys 1:400, capuchin monkeys 1:1000).

Some samples were not analyzed due to contamination or insufficient amount of saliva. In total, 114 capuchin monkey saliva samples and 341 squirrel monkey saliva samples were analyzed in duplicates using the in-house cortisol enzyme immunoassay (EIA) with an assay antibody against cortisol-3-CMO:BSA. This EIA demonstrated the following cross-reactions: cortisol, 100%; cortisone, 9.6%; corticosterone, 6.2%; 5α-dihydrocortisol, 4.6%; 5α-tetrahydrocortisol, 0.8%; 5β-tetrahydrocortisol, 0.1%; several other 5β-reduced metabolites, <0.01%. For a more detailed description of the assay please refer to Palme and Möstl [54].

For the capuchin monkeys, the intra- and inter-assay coefficients of variation were 8.1% and 10.8%, respectively. In the squirrel monkeys, the intra-assay coefficient was 7.7% and the inter-assay coefficient was 8.6%.

### 2.4. Biochemical Validation

To characterize the substances measured using the selected cortisol EIA, we performed a reversed-phase high-performance liquid chromatography (RP-HPLC) on Novapak C18 columns (3.9 × 150 mm, Millipore Corporation, Milford, MA, USA) with a Mini-Guard column (C18 Guard-Pak TM, WAT085825, Waters Corporation, Milford, MA, USA) used at room temperature with the following linear gradient: 30% to 50% methanol/water over 30 min. The flow rate was 1 mL per min and three fractions per minute were collected [58]. One pool sample (mix of aliquots of samples collected in 2013) for each species was used for this analysis. The immunoreactivity was measured in each fraction using the cortisol EIA (see [54]). The RP-HPLC analysis was conducted at the Department of Biomedical Sciences of the University of Veterinary Medicine, Vienna, Austria.

Furthermore, we pooled 10 capuchin monkey and 10 squirrel monkey saliva samples and serially diluted the pools with assay buffer to assess parallelism. The capuchin monkey samples pool was diluted as follows: 1:10, 1:50, 1:100, 1:200, 1:300, 1:400, 1:500, 1:1000, 1:10,000 and 1:50,000. The squirrel monkey samples pool was diluted as follows: 1:100, 1:200, 1:300, 1:400, 1:450, 1:500, 1:550, 1:600, 1:700, and 1:800.

### 2.5. Statistical Analyses

To investigate the daily variation pattern in cortisol secretion (within the two-time frames of the research slots) we constructed linear mixed-effect models for each species. To achieve a more symmetrical distribution, we log-transformed the response variable in cortisol concentration. Sampling time in minutes (since midnight) was included as the predictor variable and the age of the individual (in days) at the time of sampling as the control variable. As individuals may vary regarding their salivary cortisol excretion profiles and potential group effects on the salivary cortisol levels, random intercepts for individual (ID) and group were included in the models. However, due to a singularity issue resulting from the lack of variance accounted for by the group variable, the group was excluded from the model in both species.

All statistical analyses were performed using R version 3.5.2 [59]. The mixed models were constructed using the *lmer* function of the *lme4* package [60] and fitted with maximum likelihood estimation. *p*-values were generated using the *lmerTest* package [61]. Figures were created using the *ggplot* package [62]. To ensure the absence of collinearity issues, we inspected variance inflation factors (VIF: squirrel monkeys = 1.015, capuchin monkeys = 1.010) using the *vif* function of the *car* package [63]. Diagnostic plots of model residuals were examined to identify any potential violations of the normality assumption and homogeneity of variance. Initially, the residuals of the model for the capuchin monkeys were not normally distributed. To ensure normal distribution of the residuals, two data points with deviating residuals were removed from the data set.

## 3. Results

### 3.1. Biochemical Validation (HPLC and Parallelism)

**Figure 2:**
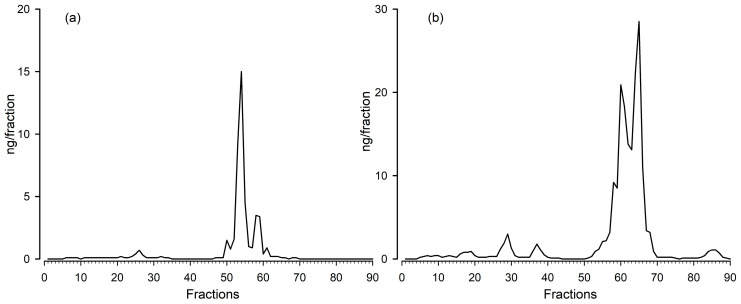
High-performance liquid chromatography (reverse phase) separation of immunoreactive substances in a salivary pool sample of (**a**) squirrel monkeys and (**b**) capuchin monkeys when measured with the cortisol EIA.

Squirrels and capuchin monkeys showed very similar profiles of immunoreactivity. In both species, two main peaks were identified (Figure 2), which showed immunoreactivity with the cortisol EIA. The first peak eluted at the approximate position of the cortisone standard; the second at the position of cortisol. Unfortunately, the pool samples of the two species were analyzed at different times and different HPLC columns were used for the separation. Therefore, the standards (and thus also the immunoreactivity) eluted at slightly different positions (later in case of capuchin monkeys).

**Figure 3:**
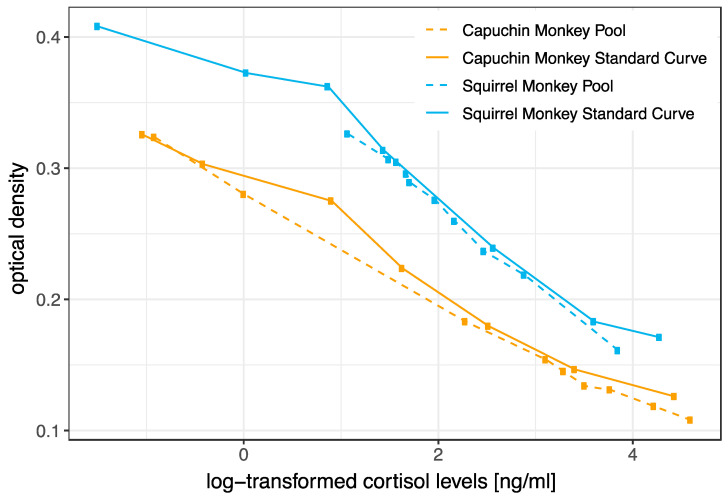
Parallelism between the standard curve (solid lines) and serial dilutions of salivary cortisol (dashed lines) of capuchin monkeys (orange lines) and squirrel monkeys (blue lines). The *y*-axis shows the optical density, the *x*-axis shows log-transformed levels of cortisol [ng/mL]. For both species, standard curves and serial dilution curves are parallel to each other.

Serially diluted pooled saliva samples were parallel to the cortisol standard curve in both capuchin and squirrel monkeys (Figure 3).

### 3.2. Daily Pattern in Cortisol

**Figure 4:**
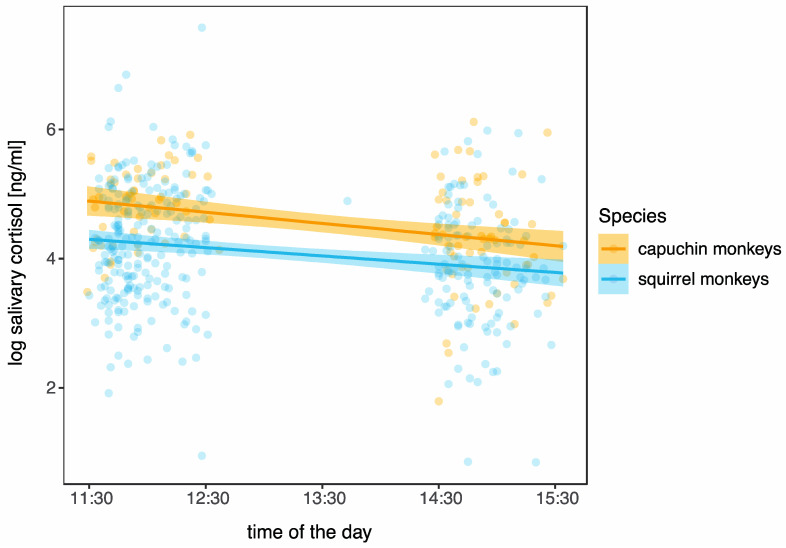
Diurnal patterns of salivary cortisol levels (log-transformed) plotted against time of the day for capuchin monkeys (orange) and squirrel monkeys (blue). Individual dots represent individual data points. The lines are linear regression lines with 95% confidence intervals as calculated from the model. Salivary cortisol levels decrease throughout the day in both capuchin and squirrel monkeys, as expected due to the circadian rhythm in salivary cortisol excretion patterns.

Overall, salivary cortisol levels in squirrel monkeys ranged between 2.3 and 1957.5 ng/mL; and 3.0 and 454.8 ng/mL in capuchin monkeys. Table 1 gives an overview of salivary cortisol level measures by species. Linear mixed effect models found that cortisol concentrations in both species declined in the course of the day (Table 2, Figure 4).

## 4. Discussion

To the best of our knowledge, our study represents the first investigation to measure salivary cortisol in capuchin monkeys (*Sapajus* spp.) and a group of female squirrel monkeys (*Saimiri sciureus*). Additionally, it is the first study to utilize an EIA for the measurement of salivary cortisol in the two monkey species. Previous studies on salivary cortisol in squirrel monkeys (male or sex not reported) have employed different methods, such as radioimmunoassays (RIAs; [49,50,51,52,53]) or time-resolved fluorescence detection (DELFIA; [48]) for cortisol determination. Our findings demonstrate that the two species have salivary GCs and that they can be reliably measured using cortisol EIA.

The immunograms obtained from the HPLC and the EIA using antibodies raised against cortisol-3-CMO:BSA [54] confirmed that saliva of both species contained cortisol. Excluding the peaks of cortisol, the RP-HPLC immunograms showed another peak (even dominating in squirrel monkeys) appearing somewhat earlier that approximately corresponded with the position where cortisone eluted (cross-reactivity = 9.6%). Cortisone was also detected previously in the feces of capuchin monkeys [46]; in the urine of marmoset monkeys [64] using the same EIA; and in the blood of other non-human primates (e.g., [65,66]). Due to its cross-reactivity with the cortisol EIA, the presence of cortisone in these New World primates’ saliva should not be entirely overlooked. Several studies found cortisol as well as cortisone in human saliva (e.g., [67,68,69]). Meulenberg and colleagues [68] reported that cortisone and cortisol levels correlate in saliva as well as plasma in humans. Furthermore, cortisone follows a circadian pattern that runs parallel to the rhythm of cortisol and that can be detected in saliva, urine, and blood of human subjects [69,70]. Cortisone levels in human saliva were higher than cortisol concentrations [67,69], which was attributed to the activity of an enzyme in the parotid glands that oxidizes cortisol to cortisone [67,68]. Similar patterns and relationships between cortisone and cortisol levels were found in the saliva and might be expected in the blood of capuchin and squirrel monkeys. On the one hand, this calls for further studies exploring levels of cortisone (in relation to levels of cortisol) in salivary samples of these two primates. On the other hand, it suggests that cortisone should be considered in study designs and that the measurement of cortisone might be an alternative in future studies (e.g., cortisone levels should indeed be higher but correlated to cortisol levels in saliva of these monkey species, required amounts of saliva could (for instance) be reduced).

Furthermore, we validated our assay by showing that concentrations of serially diluted pooled saliva samples were parallel to the cortisol standard curves in both species. This suggests that the assay reliably measures cortisol levels in the saliva of both New World monkey species. An ACTH (adrenocorticotropic hormone) challenge, which is a commonly used physiological validation method [34], was not performed in the scope of the current study as no invasive procedures were performed. Therefore, we aimed to assess the biological suitability of the selected method for analyzing salivary cortisol levels in the two New World primate species by determining relevant changes in salivary cortisol levels, i.e., we investigated whether salivary cortisol levels collected during the morning and afternoon research sessions corresponded to the species’ diurnal secretion pattern of cortisol observed in blood samples [55,56]. Due to practical constraints, such as limited data collection times at Edinburgh Zoo, we were unable to examine the entire circadian cortisol excretion pattern. Nevertheless, our findings demonstrate that, as expected, daytime influenced salivary cortisol levels, with higher levels observed earlier in the day. This confirms that using the tested EIA allows for tracking biologically relevant changes in salivary cortisol levels in both species. Furthermore, many New World primate species were reported to have particularly high levels of (salivary) cortisol levels [71,72], which was confirmed in our study, i.e., salivary cortisol levels in squirrel monkeys and capuchin monkeys were markedly higher than those reported for bonobos, gorillas, and orangutans in the study [27] where saliva samples of these apes were measured with the same assay as employed in the current study.

## 5. Conclusions

In summary, we successfully validated the in-house cortisol enzyme immunoassay (EIA) for measuring salivary cortisol levels in capuchin monkeys and squirrel monkeys. Our results provide valuable information on the applicability of the employed method and can be informative for researchers interested in studying salivary cortisol in these New World primate species (e.g., [73]). Further research exploring the relationship between cortisol and cortisone levels in the species’ saliva is recommended to enhance our understanding of their hormonal profiles and stress responses.

## Figures and Tables

**Table 1:** Descriptive statistics of salivary cortisol levels in capuchin monkeys and squirrel monkeys.

				Salivary Cortisol [ng/mL]					
	Sex	Individuals	Samples	Min.	1st Qu.	Median	Mean	3rd Qu.	Max.
Squirrel monkeys	females	17	341	2.33	33.38	61.35	96.13	119.08	1957.52
Capuchin monkeys	males	8	114	3.00	60.38	95.25	123.77	179.18	454.80

**Table 2:** Results of the linear mixed effects model of the squirrel monkeys and the capuchin monkeys. The values given are estimates, standard errors (SE), confidence intervals (CI), degrees of freedom (df), t-values, and *p*-values. *** *p* < 0.01.

	Parameter	Estimate	SE	CI	df	t	*p*	
Squirrel monkeys	(Intercept)	5.996	0.516	(4.980, 7.041)	158.700	11.621	<0.001	***
	time of day	−0.002	0.001	(−0.003, −0.001)	328.000	−3.600	<0.001	***
	age (in days)	0.000	0.000	(0.000, 0.000)	13.140	−0.739		
Capuchin monkeys	(Intercept)	7.313	0.666	(5.978, 8.631)	68.700	10.987	<0.001	***
	time of day	−0.003	0.001	(−0.004, −0.002)	104.700	−4.488	<0.001	***
	age (in days)	0.000	0.000	(−0.001, 0.000)	7.999	−0.836		

## Data Availability

Data are available on request.
